# Discriminative Random Field Segmentation of Lung Nodules in CT Studies

**DOI:** 10.1155/2013/683216

**Published:** 2013-07-02

**Authors:** Brian Liu, Ashish Raj

**Affiliations:** ^1^Cornell University, Ithaca, NY 14853, USA; ^2^Weill Cornell Medical College, New York, NY 10065, USA

## Abstract

The ability to conduct high-quality semiautomatic 3D segmentation of lung nodules in CT scans is of high value to busy radiologists. Discriminative random fields (DRFs) were used to segment 3D volumes of lung nodules in CT scan data using only one seed point per nodule. Optimal parameters for the DRF inference were first found using simulated annealing. These parameters were then used to solve the inference problem using the graph cuts algorithm. Results of the segmentation exhibited high precision and recall. The system can be adapted to facilitate the process of longitudinal studies but will still require human checking for failed cases.

## 1. Introduction

 Traditionally, the analysis of tumors through computed tomography (CT) scans involved time consuming manual segmentation of tumor volumes, where a radiologist or technician would draw ROIs encapsulating the tumor areas by hand. Numerous semiautomatic segmentation algorithms have been proposed for a variety of tumors, including brain [1], liver [2], breast [3], and lung [4]. In certain cases, such as Zhang et al. [1], the proposed method was not specific to a certain kind of tumor. In other cases, such as Kostis et al. [4], the segmentation required prior knowledge about the characteristics of the types of tumors observed in order to do morphological processing.

There exists a significant opportunity for reducing the human input required for nodule segmentation in longitudinal studies. An initial seed point given at the first time point can be coregistered and extrapolated to subsequent studies, under the assumption that nodules do not exhibit significant movement. This is particularly useful in a clinical application for tracking small pulmonary nodules in the lungs to determine malignancy [4].

Markov random fields (MRFs) have been used in the area of computer vision for segmentation by solving an energy minimization problem [5]. We use the pixel grid as a graph, in which each pixel is a vertex and neighboring pixels share an edge between them. We can then define an energy cost for any given labeling as a function of various features of the MRF. In the traditional MRF definition, the energy potential can be expressed as an association potential function of each node and an interaction potential function of pairs of neighbors. The goal is then to find an optimal labeling which minimizes the total energy. Solving the inference problem afterwards can be done quickly and optimally (for binary labels, for multiple label and within an approximation factor) using an optimization method such as graph cuts [6–9]. Picking the right potential functions can often be a matter of trial and error.

There are several variants of MRFs out in the literature. In particular, conditional random fields (CRFs) generalize the MRF formulation by allowing data to factor into the traditional MRF interaction potential formulation, with a discriminative model instead of a generative model. Kumar and Hebert's discriminative random fields (DRFs) [10] extend the usual work of conditional random fields to multiple dimensions. In particular, Kumar and Hebert's construction allows for the use of a variety of discriminative models, like SVMs [11].

DRFs do suffer from some problems, however. Because the learning process uses a pseudolikelihood approximation, the results tend to overestimate the interaction potential parameters, unless careful regularization is used [10]. We avoid this issue by optimizing using simulated annealing on the F-score, so that inference results play a direct role in the optimization. The F-score is a direct measure of inference performance, so optimization based on the F-score should give us better results than pseudo-likelihood maximization. Unfortunately, this sacrifices many of the nice properties of the original formulation, such as convexity. In practice, however, F-score optimization consistently produces slightly better results. This method has been tried before for CRFs, with better reported performance than standard CRF training [12].

Our goal in this paper is to apply DRF methodology to the segmentation of lung nodules in CT scans. To our knowledge, this has never been attempted before. A recent work by Ye et al. [13] has used graph cuts to segment lung nodules but did not use an underlying discriminative model to train their energy function. DRFs have been used by Lee et al. [11] for brain tumors in MRI scans, with good results. The DRF methodology provides a strong, flexible framework for image segmentation tasks that provides more robust segmentations than nongraphical models. For example, a previous study by Kostis et al. has demonstrated a successful lung nodule segmentation algorithm through thresholding and morphological processing [4] that required identification of nodule type (e.g., juxtapleural, juxtavascular). This followed, earlier work by Zhao et al. using progressive thresholding and a conditional shape constraint [14]. These methods require different parameters for different kinds of nodules, which makes the job of segmentation more time consuming. More recently, Hayashi et al. used thresholding and morphological filtering to accomplish the same goal [15]. While morphological filtering can do well at estimating volumes, the filters often smooth away surface data, which has to be restored via some other method.

On the other hand, Xu et al. used dynamic programming and expectation maximization to calculate the optimal boundaries of lung nodules, using a shape constraint to counter the problem of juxtapleural nodules [16]. This method avoids the problem of smoothing away surface data but does not always perform well, requiring human intervention. In addition, Xu et al. work on each slice independently, which does not take advantage of the spacial information from working in three dimensions. Similarly, a work has been done by Okada et al. on robust 2D ellipsoid fitting on synthetic data [17], though their work does not focus on the end segmentation.

Using DRFs, we can incorporate simpler, more approximate morphological filtering into a set of other features and pairwise constraints to achieve an overall more accurate and robust segmentation. Coming up with good features is rarely a systematic process; instead one must often rely on intuition and human knowledge of the problem. In the case of lung nodules, it is known that a lung nodule is generally located around its seed point, has CT intensities in a certain range, and is usually round [18]. This paper shows that good results can be achieved even with simple features containing this information. Furthermore, we can easily learn parameters from training data and test performance on test data to avoid the risk of overfitting. The DRF framework allows us to swap out features as we see fit, giving us the ability to adapt the method for other volumes that need segmentation. Since the ultimate goal of this research is to create a semiautomatic segmentation algorithm that can be applied to other types of tumor segmentation tasks, this is a great advantage.

## 2. Materials and Methods

### 2.1. Data

 The data set consisted of 4 pairs of training nodules and 50 pairs of testing nodules from the VOLCANO'09 Challenge [19]. For training and individual results, only the first of each pair was used. For longitudinal comparison results, we numbered each individual nodule such that nodules *x* and 50 + *x* are the first and second nodules in pair *x*, respectively. These numbers will be used throughout. Seed points were given with the data sets. Training was done on the supplied training set only, with results evaluated on the supplied testing set only.

The training set nodules showed variation in image noise but lacked variation in nodule position. In particular, the training set contained no juxtapleural or juxtavascular nodules. These kinds of nodules do show up in the testing set. In order to maintain consistency with the VOLCANO'09 Challenge, however, the training and test sets were not rearranged.

Ground truth voxel labelings for all nodules were done manually by a graduate research fellow trained by a radiologist.

### 2.2. Algorithm Summary

 Several features, such as estimated radius and approximate segmentation, are first calculated through a morphological filtering process. We will then use supervised learning to learn the weights for these features in a DRF model of lung nodules from labeled training scans. The details of the feature generation and parameter learning are described in the following section. After we have learned the parameters, we can solve the inference problem using the same feature generation process and graph cuts to obtain a segmentation on new scans.

#### 2.2.1. Constants and Nodule Feature Extraction

**Figure 1:**
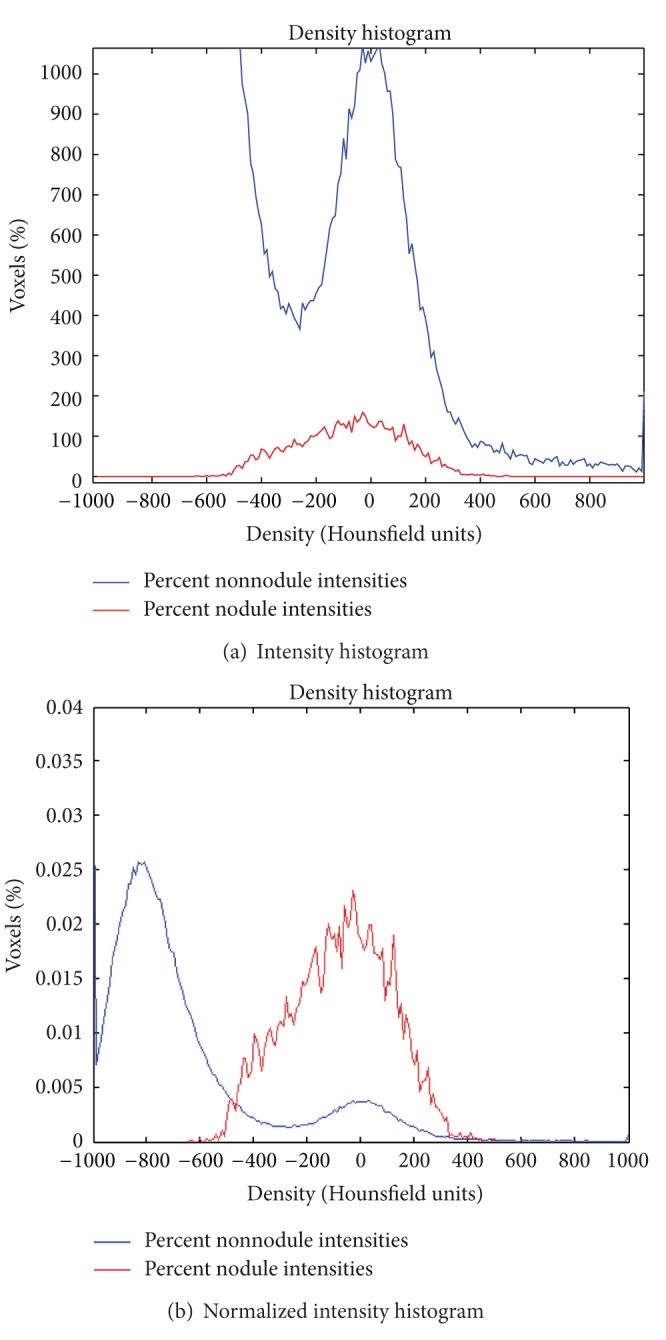
(a) Histogram of voxel intensities of positive examples (nodule voxels) and negative examples (everything else) in training data. Negative examples overwhelm positive examples in all intensities, even in the local area, as shown in this histogram. We must thus exploit locality to achieve a good segmentation. (b) Histogram normalized by number of voxels.

 We first calculate several global constants from the data. A Gaussian model of nodule voxel intensities was calculated from the training data with constants *μ*_int⁡_ and *σ*_int⁡_ for the mean and standard deviation, respectively. A uniform model (threshold model) was calculated from the training data with constants *t*_min⁡_, *t*_max⁡_ as the minimum and maximum thresholds. As seen in Figure 1, a Gaussian distribution can fit the nodule voxels to a first approximation.

In addition, for each nodule, its radius was estimated by taking the following steps.

1. Denoising: an in-slice Gaussian filter of one voxel standard deviation was applied to smooth out high frequency noise, and then upper and lower thresholds were applied to obtain an initial segmentation.
2. Subvolume and initial radius estimation: a rough estimate of radius *r*_init_ was obtained by growing a bounding box and stopping when the fraction of voxels not in the initial segmentation reached 0.75 of the total volume.
3. Lung subvolume extraction: a morphological close followed by a morphological open operation with an anisotropic sphere with 6 mm radius (under the assumption that most features in the lung are smaller than 6 mm) was performed on the inverse of the initial segmentation. The nodule area was filled in with an anisotropic sphere of radius *r*_init_/2 centered at the input point, and a morphological close operation was applied to arrive at the final lung volume.
4. The initial segmentation was filtered to only include voxels in the lung volume and filtered again to only include the voxels in the same connected component as the seed point.
5. The center of the nodule was recalculated by finding the local maximum of the 2D distance transform (distance from outside the smoothed segmentation) closest to the seed point on the same slice.
6. The final estimated nodule radius *r* was calculated by expanding a sphere from the new center until we included no more segmented voxels or the fraction of smoothed segmentation voxels inside the sphere reached less than 0.5.

### 2.3. DRF Framework

 We construct a DRF model of the CT volume as follows.

Let *G* = (*S*, *E*) be the graph that represents the 3D volume, where each node in *S* represents a voxel and an edge in *E* connects adjacent voxels in a 6-neighborhood. Let *n*_*i*_ be the observed intensity at voxel *s*_*i*_ ∈ *S*, let *p*_*i*_ be the 3-vector of the relative coordinates of voxel *s*_*i*_ in the volume, and let label *x*_*i*_ ∈ {−1,1} be the label associated with *s*_*i*_. We define an observation *y*_*i*_ = (*n*_*i*_, *p*_*i*_). The random variables *x*_*i*_ obey the Markov property that Pr(*x*_*i*_ | *y*, *x*_*Si*_) = Pr(*x*_*i*_ | *y*, *x*_*N*_*i*__), where *N*_*i*_ is the set of neighbors of *s*_*i*_ and *Si* is everything in *S* except *s*_*i*_.

Assuming only pairwise clique potentials to be nonzero.

(1) $$\begin{array}{c} \text{Pr}\left(x\;|\;y\right)=\frac{1}{Z}\exp\left(\underset{i\in S}{\sum }A_{i}\left(x_{i},y\right)+\underset{i\in S\;}{\sum }\underset{\;j\in N_{i}}{\sum }I_{ij}\left(x_{i},x_{j},y\right)\right), \end{array}$$

where *Z* is the partition function, *A*_*i*_ is an association potential, and *I*_*ij*_ is an interaction potential.

#### 2.3.1. Association Potential

 We model the association potential discriminatively using a logistic model since the labels are binary. We will define a feature vector *f*_*i*_ at site *s*_*i*_ as a function of the observations *y*. The location of the lung nodule voxels was also modeled as a Gaussian deviating from a prior known location normalized by the estimated nodule radius *r*, calculated automatically, and constants *l* = (*l*_*x*_, *l*_*y*_, *l*_*z*_) and *σ*_loc⁡_ = *d*/*v*, where *v* is the size of the voxel in *x*, *y*, and *z* physical coordinates.

We then define our feature vector to be

(2) $$\begin{array}{c} f_{g}=\sqrt{\frac{{\left(n_{i}-\mu \right)}^{2}}{\sigma^{2}}},\\ f_{u}=\{\begin{array}{cc} 0, & \text{if}\;\;n_{i}>t_{\min}\wedge n_{i}<t_{\max},\\ 1, & \text{otherwise}, \end{array}\\ f_{l}={||\frac{\left(p_{i}-l\right)}{\sigma_{\mathrm{loc}}}||}_{2},\\ f_{i}\left(y\right)=\left[f_{g},f_{u},f_{l}\right]. \end{array}$$

The first two features capture the cost of a voxel's intensity in a Gaussian model and a uniform model, respectively. The third feature captures the cost for a distant voxel from the expected nodule center.

We then have the option of transforming our feature vector via some nonlinear transformation to *h*_*i*_(*y*) = [1,*ϕ*_1_(*f*_*i*_(*y*)),…,*ϕ*_2_(*f*_*i*_(*y*))]^*T*^, which is a kernel mapping of our original feature vector with the introduction of a bias element. We chose not to use a kernel, so *ϕ*(*f*_*i*_(*y*)) = *f*_*i*_(*y*). The features are then weighted by a parameter *w*.

We formulate our association potential as a probability by applying a logistic function

(3) $$\begin{array}{c} \text{Pr}\left(x_{i}=1\;|\;y\right)=\frac{1}{1+e^{-w^{T}h_{i}(y)}}. \end{array}$$

Since Pr(*x*_*i*_ = −1 | *y*) = 1 − Pr(*x*_*i*_ = 1 | *y*), we can express this probability more compactly as

(4) $$\begin{array}{c} \text{Pr}\left(x_{i}\;|\;y\right)=\frac{1}{1+e^{-x_{i}w^{T}h_{i}(y)}}. \end{array}$$

Finally, we model the association potential as the log of this probability in order to preserve the logistic regression characteristics when the interaction potential factor is zero [10]:

(5) $$\begin{array}{c} A\left(x_{i},y\right)=\log\left(\frac{1}{1+e^{-x_{i}w^{T}h_{i}(y)}}\right). \end{array}$$

The parameter to learn in the association potential is then *w*.

#### 2.3.2. Interaction Potential

 We model the interaction potential using the pairwise smoothing of the Ising model, normalized by a constant minus the difference in intensities of the two sites. We will define a new feature vector *δ*_*ij*_(*y*) that captures this difference:

(6) $$\begin{array}{c} \delta_{ij}\left(y\right)={\left[\max\left(\frac{1-\left|n_{i}-n_{j}\right|}{1000,0}\right)\right]}^{T},\\ I\left(x_{i},x_{j},y\right)=\beta \left(x_{i}x_{j}v^{T}\delta_{ij}\left(y\right)\right). \end{array}$$

The *β* term is a constant term controlling whether the smoothing cost affects the potential. The parameter to optimize, then, is *v*.

### 2.4. Learning and Inference

#### 2.4.1. Performance Metrics

 The primary performance metrics for evaluation used are precision and recall. Given a calculated labeling *O* and the ground truth labeling *G*, where nodule voxels are positive samples and nonnodule voxels are negative, tp denotes true positive, fp denotes false positive, and fn denotes false negative. Precision and recall are then defined as

(7) $$\begin{array}{c} \text{precision}=\frac{\text{tp}}{\text{tp}+\text{fp}},\\ \text{recall}=\frac{\text{tp}}{\text{tp}+\text{fn}}. \end{array}$$

#### 2.4.2. Learning

Optimal parameters were learned using simulated annealing on the F-score of inference results on training data.

Given parameters *θ* = (*w*, *v*), there exists an optimal label *O* such that, for each *x*_*i*_ given *y*, *A*(*x*_*i*_, *y*) + ∑_*j*∈*N*_*i*__ *I*(*x*_*i*_, *x*_*j*_, *y*) is greater than *A*(*x*_*i*_, *y*) + ∑*j* ∈ *N*_*i*_ *I*(*x*_*i*_, *x*_*j*_, *y*) (where *x*_*i*_ denotes the opposite label of *x*_*i*_). The optimal labeling is calculated using graph cuts [5].

Optimal parameters were found by performing simulated annealing on the F-score function, defined as 2(precision∗recall/(precision + recall)). At a given iteration *i*, a segmentation was calculated with graph cuts using parameters *θ*_*i*_ generated randomly from the previous parameters *θ*_*i*−1_, constrained distancewise by a “temperature” parameter that slowly decays as the iterations increase. The calculated segmentation is then used to calculate the F-score, which is compared to the F-score of the previous iteration as part of the simulated annealing process. Matlab's simulated annealing implementation was used to find the optimal parameters. Boundary parameters were (−Inf, Inf) for all parameters in *θ*. Initial parameters for simulated annealing were $\theta =\vec{0}$. After the initial run, boundary parameters were picked by hand to include the optimum with tighter one-sided bounds to improve running time for subsequent runs. This did not change the optimum parameter appreciably, so the initial parameters were changed to the optimum parameters. Again, this did not change the optimum parameters upon rerunning simulated annealing. This gives us more confidence that the optimum parameters we found are in fact optimal in its local neighborhood.

#### 2.4.3. Inference

The volume was first smoothed with a one voxel radius Gaussian filter to get rid of high frequency noise. An exact maximum a posteriori solution was then obtained for the pairwise Ising model by a graph cuts algorithm. Graph cuts were performed using Olga Veksler's gco-3.0 library in C++ with a Matlab wrapper [6, 9].

## 3. Results

### 3.1. Segmentation

**Figure 2:**
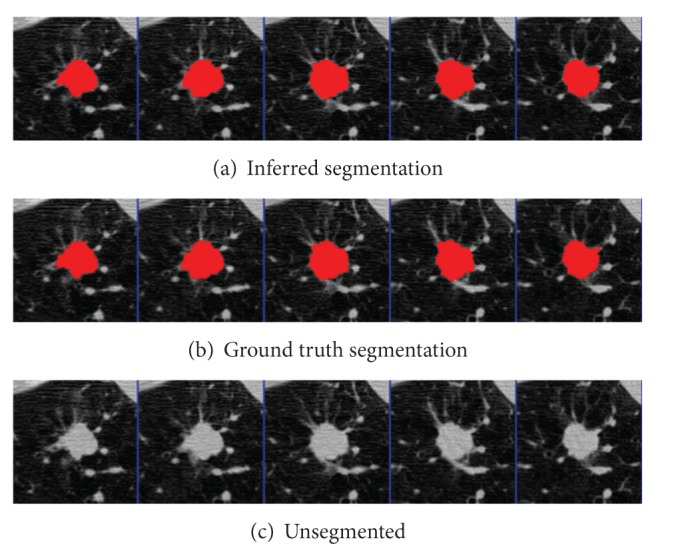
Tumor 11: comparison of inferred segmentation versus the ground truth labeling with the unsegmented subvolume for reference.

**Figure 3:**
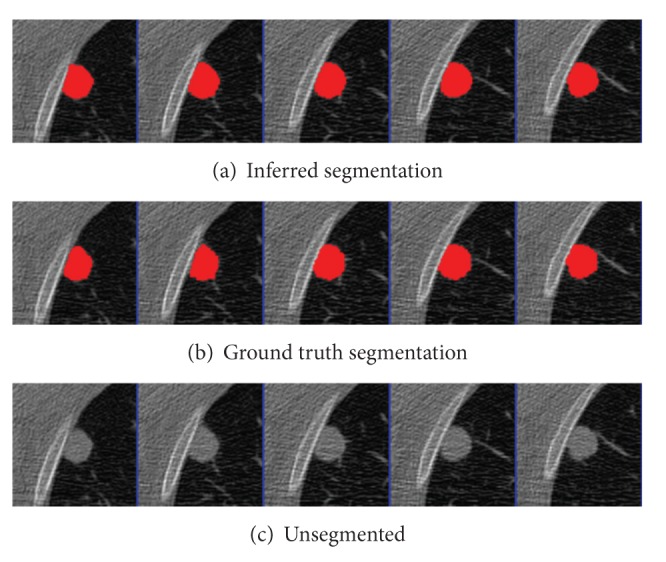
Tumor 23: Comparison of inferred segmentation versus the ground truth labeling with the unsegmented subvolume for reference.

 The parameters were learned from the first nodules of the 4 given pairs of training nodules. Results were segmented using graph cuts on the first nodules of the 50 pairs of test nodules. The mean precision was 0.92 and the mean recall was 0.89, not accounting for the size of the nodules. An example segmentation and the ground truth can be seen in Figures 2 and 3. When all 50 pairs (100 nodules) were evaluated, the mean precision was 0.91 and the mean recall was 0.89.

**Figure 4:**
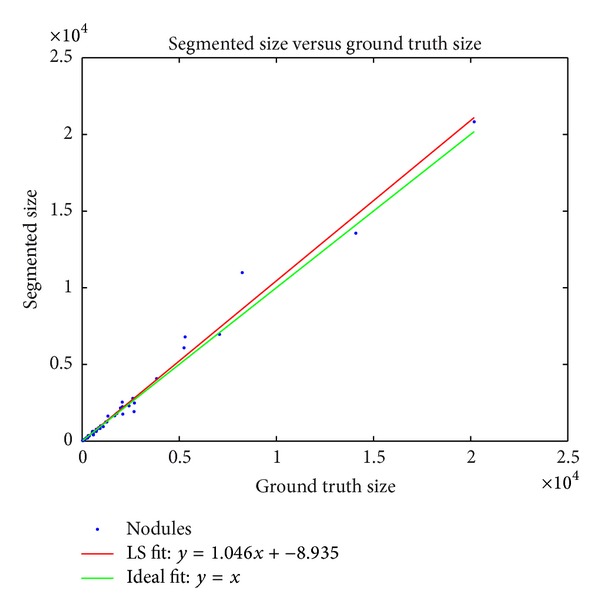
Plot of segmented volume size versus ground truth volume size. An ordinary least squares fit is shown, along with the expected fit, *y* = *x*. The correlation coefficient *R* = 0.99, and the *P* value *P* = 0.00. Our method accurately estimates the volumes compared to ground truth with no significant bias towards either a larger or a smaller segmentation.

The segmented physical volumes were plotted against the ground truth physical volumes in Figure 4. An ordinary least squares fit was applied to the data, and the fit line closely approximates the expected fit line, *y* = *x*. The correlation coefficient *R* = 0.99, and the *P* value *P* = 0.00. This shows that our method accurately estimates the volumes compared to ground truth and that there is no significant bias towards either a larger or a smaller segmentation.

**Figure 5:**
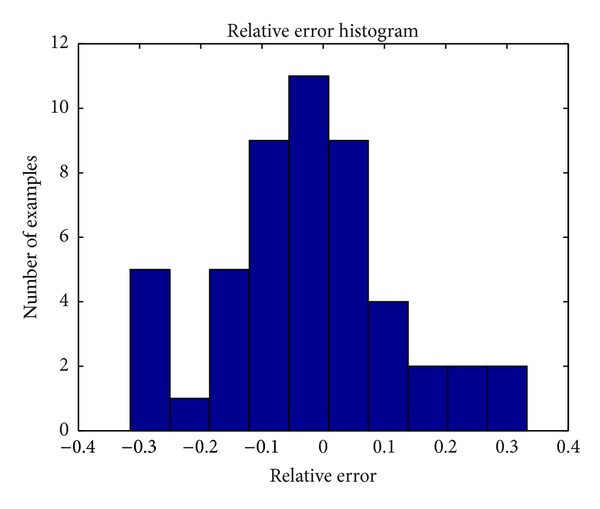
Histogram of errors relative to ground truth volume.

The relative volume error compared to ground truth was calculated for each of the first 50 test examples. The maximum positive error was 0.33 and the maximum negative error was −0.31. A histogram of the relative errors is shown in Figure 5.

**Figure 6:**
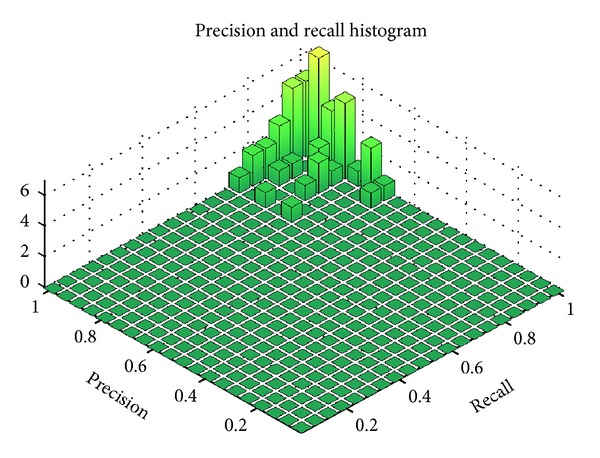
Histogram of precision and recall of first 50 segmented examples.

A 2D histogram of the precisions and recalls is shown in Figure 6. Most examples had precisions and recalls within the 0.8 to 1.0 range.

**Figure 7:**
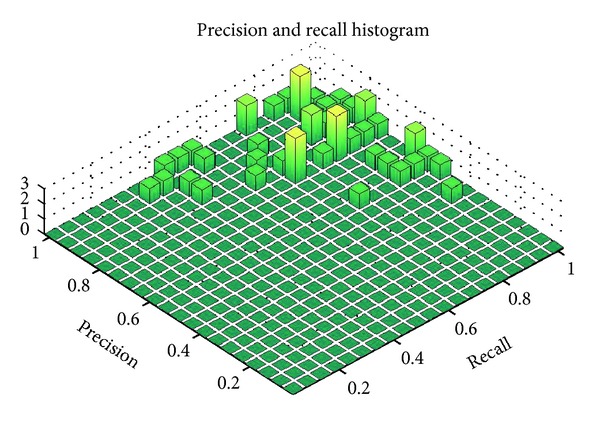
Histogram of precision and recall of first 50 Slicer RSS segmented examples.

**Figure 8:**
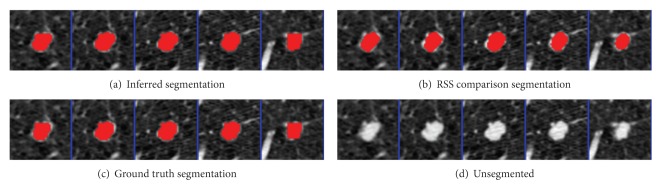
Tumor 20: comparison of inferred segmentation versus RSS, ground truth, and the unsegmented subvolume for reference. In this example, RSS overestimated the roundness and undersegmented the nodule. Our method successfully segmented the bumps.

**Figure 9:**
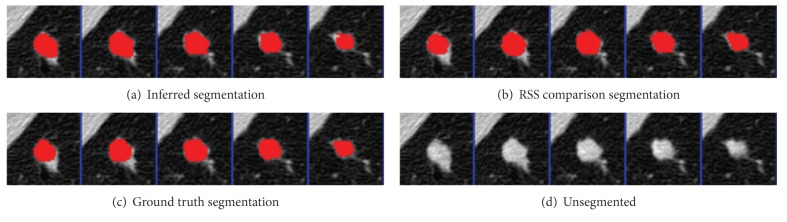
Tumor 40: comparison of inferred segmentation versus RSS, ground truth, and the unsegmented subvolume for reference. In this example, both segmentation methods performed well.

**Figure 10:**
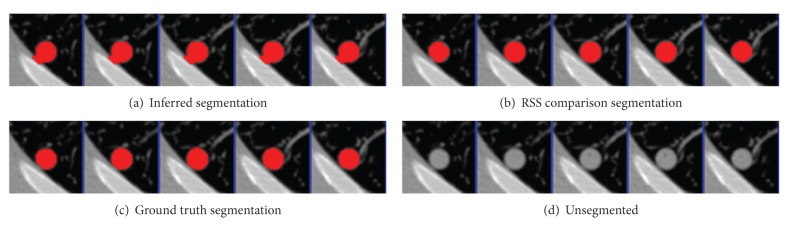
Tumor 50: comparison of inferred segmentation versus RSS, ground truth, and the unsegmented subvolume for reference. This is an example in which our method oversegmented into the pleural wall, while RSS did not.

**Figure 11:**
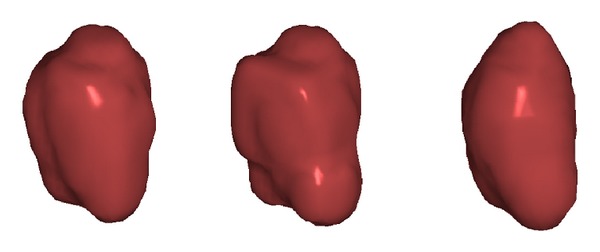
Tumor 20: comparison of our segmented volume versus RSS and ground truth for reference. From left to right: our segmented volume, ground truth volume, and RSS segmented volume.

**Figure 12:**
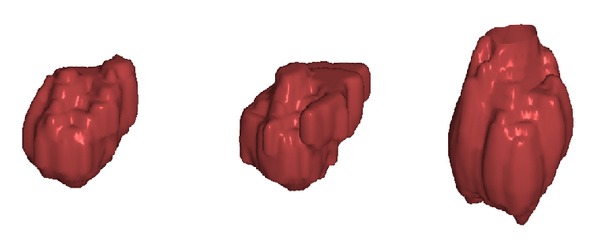
Tumor 30: comparison of our segmented volume versus RSS and ground truth for reference. From left to right: our segmented volume, ground truth volume, and RSS segmented volume. Both segmentation methods performed poorly, but RSS vastly oversegmented the nodule compared to our method.

**Figure 13:**
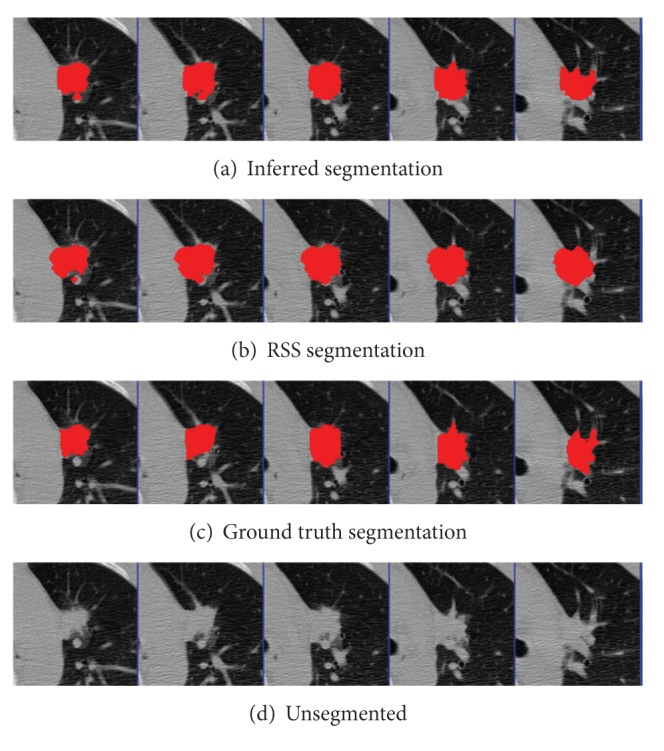
Tumor 30: comparison of inferred segmentation versus RSS with ground truth and unsegmented subvolume for reference.

As a comparison test, performance was compared to the Robust Statistical Segmentation procedure implemented in Slicer. The RSS method uses a statistics-driven active contour model for segmentation [20]. Approximate volumes were specified using ground truth data. Boundary and intensity uniformity parameters were tuned by hand for each nodule until a satisfactory or best possible segmentation was achieved. Slicer RSS achieved a mean of 0.78 precision and 0.78 recall under these conditions. A histogram of the results can be seen in Figure 7. RSS is more inconsistent with its performance compared to our method. Some segmentations can be seen in Figures 8, 9, and 10, and a volume rendering can be seen in Figure 11. As a whole, our method performed better than RSS used by Slicer, but in some individual cases like Figure 10 RSS performed better. There are examples in which both methods performed poorly as well: Tumor 30 is such an example, largely due to significant vascularization of the nodule and its juxtapleural position. A volume rendering comparison of Tumor 30 can be seen in Figure 12. RSS oversegmented the nodule significantly, while DRF also oversegmented the nodule to a lesser extent. A slice-by-slice comparison can be seen in Figure 13.

**Figure 14:**
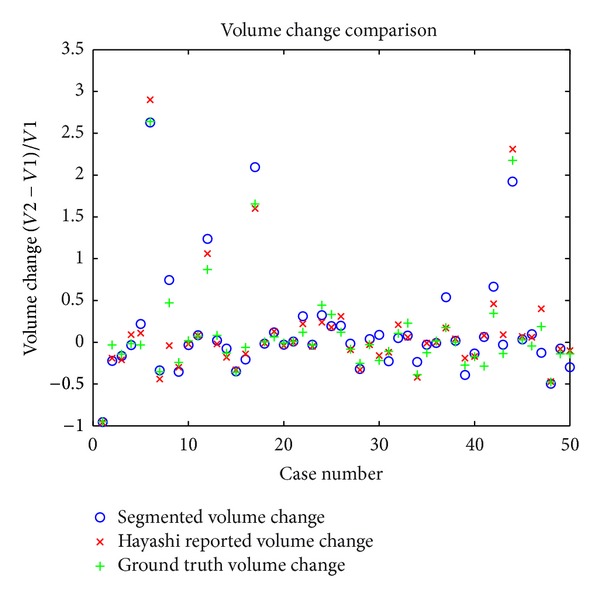
Percent volume change versus Hayashi et al.'s percent volume change and our ground truth percent volume change.

The metric used to evaluate performance in the VOLCANO'09 Challenge is percent volume change (*V*2 − *V*1)/*V*1 from the first sample volume of a pair (*V*1) to the second one (*V*2). In Figure 14, the percentage change for each testing pair was plotted against the percentage change from a participant [15] and against the percentage change of our ground truth. Because there was no previous ground truth percentage change established for the challenge, our ground truth does not reflect the desired results of the challenge.

## 4. Discussion

 Due to the lack of widely available dedicated lung nodule segmentation software currently, it is difficult to compare our results with existing standards. In comparison with similar work, Ye et al. report a mean Dice's coefficient of 0.79 on 101 nodules [13]. Our Dice's coefficient (which is an equivalent definition to the F-score in this context) is 0.90. The standard deviations of our F-scores were both around 0.06. We suspect that our superior performance despite simpler features can be explained by two factors: first, our discriminative model and training gave us a better energy function; and second, simpler metrics may prove to be more tolerant to error. Dehmeshki et al. did not do a voxelwise comparison but instead reported an “acceptability” metric of 0.84, as determined by radiologist examination [21]. Kostis et al. seemed to have achieved very good results, but they did not report explicit performance metrics comparing their results to ground truth [4]. Neither Zhao et al. [14] or Xu et al. [16] reported data sets or performance metrics compared to ground truth. The comparison with Robust Statistical Segmentation in Slicer shows our performance against a state-of-the-art generalized segmentation tool, and our method on average performs better.

One must also be wary of placing too much trust in ground truth. Manual segmentations currently in use may differ significantly between users, as Opfer and Wiemker pointed out [22]. Without a better idea of the variation in acceptable segmentations, one runs the risk of overfitting. For a case like Tumor 30 (which was challenging for both our algorithm and other comparison algorithms), the nearby vasculature and pleura may affect the accuracy of manual segmentations as well.

Several groups participated in the VOLCANO'09 Challenge [15, 18, 19], but because the challenge was focused on evaluating volume change in longitudinal studies instead of measuring volume itself, only volume change metrics were reported. Volume change metrics from our results were comparable to the results from Hayashi et al. [15]. Because aggregate results for the VOLCANO Challenge were renumbered before reporting in Reeves et al. [19], we did not compare their aggregate results. Given our established ground truth, however, we believe that the precision and recall are a better measure of our performance in general.

A natural extension of this work would be to apply the same method to segmentation of other tumors in the body. The problem of segmentation in other anatomical areas has of course been studied: for example, Lee et al.'s work involved segmenting MRI data on brain tumors, with results implying their precision and recall were around 0.8 that each [11].

The main advantage of the DRF learning framework is the automatic learning of energy function parameters for segmentation. Since all specific knowledge about the type of tumor we are looking for is learned automatically from the training examples as opposed to knowledge that is built into the algorithm, we can in theory train our model to work with other types of tumors than the lung nodules presented in this paper. In practice, lung nodules are generally easier to distinguish due to their high contrast to surrounding tissue, so applying the model to other tumors will likely produce worse results.

If the problem has been formulated properly, the theoretical optimum solution for the parameters should be the maximum likelihood solution to the DRF. Our investigation, however, found that the maximum likelihood solution favored oversegmentation, achieving a very high recall, but with losses in precision. We thus decided to use a more practical approach and optimize directly based on the metric we were using to evaluate the algorithm: the F-score, the harmonic mean of precision and recall. Our results give better recall with similar precision compared to the maximum pseudo-likelihood solution for the parameters. The difference is on the order of a few percentage points.

In practice, the inference step required to segment new nodules can be solved via fast polynomial time algorithms using graph cuts. Using unoptimized Matlab code on a 3.3 GHz quad core desktop with 8 GB RAM, this translated to sub-10 second segmentations for the volumes tested. With optimized, compiled code, this will likely be much faster.

### 4.1. Conclusion

 Our DRF semi-automatic segmentation produces results that are generally very accurate, with on average 90% precision and recall. This system can be used to facilitate lung nodule size tracking applications. Further work includes creating a clinical application in order to investigate the consistency and clinical applicability of such a system. Future work can be done to expand the algorithm's performance to different types of tumors, such as brain or liver. More consistency can be established with better radius estimation, which can be achieved through a better initial segmentation. Another possibility would be to try extending the robust ellipsoid fitting algorithm from Okada et al. [17] to three dimensions, allowing us to get a better estimate of nodule shape.
